# Effect of Temperature on Detection of Hepatitis B Surface Antigen by Gingival Swab

**DOI:** 10.7759/cureus.92883

**Published:** 2025-09-21

**Authors:** Hira Tariq, Mateen Izhar, Namra Mahmood, Aqib Sultan, Nazia Ahmad, Hadiqa tul Hafsa

**Affiliations:** 1 Pathology/Microbiology, Avicenna Medical College, Lahore, PAK; 2 Microbiology, Rahbar Medical and Dental College, Lahore, PAK; 3 Pathology, Central Park Medical College, Lahore, PAK; 4 Microbiology, Nawaz Sharif Medical College, Gujrat, PAK; 5 Pathology, Lahore Medical and Dental College, Lahore, PAK; 6 Microbiology, Shaikh Zayed Postgraduate Medical Institute, Lahore, PAK

**Keywords:** elisa, gingival swab, hbsag detection, hepatitis b, hepatitis b diagnosis, pbs solution, temperature effect

## Abstract

Objective

The objective of the study was to determine the effect of two temperature points (27-32 °C and 50 °C) on the detection of Hepatitis B surface antigen (HBsAg) by gingival swab.

Method

The study was conducted in 2024 in Shaikh Zayed Hospital, Lahore, Pakistan. It was a non-randomized controlled study done over a period of one year. The study included 138 known positive cases of hepatitis B and 138 known negative cases, as validated by serum enzyme-linked immunosorbent assay (ELISA). Patients with mouth ulcers, gum bleeding, or gingivitis were excluded. Two gingival swabs were obtained from each patient. The swabs were kept in phosphate-buffered saline solution at two different temperatures (27-32 °C and 50 °C) for 24 hours. Automated ELISA performed HBsAg detection on both samples from each patient.

Results

Out of 138 known hepatitis B positive individuals, 108 swabs tested positive for HBsAg at 50 °C and 84 at 27-32 °C. Swabs of all 138 known hepatitis B-negative patients were negative at both 50 °C and 27-32 °C.

Conclusion

HBsAg is more likely to be detected by swab samples at higher temperatures, and no cold chain is required to transport these swab samples.

## Introduction

Hepatitis B is one of the major health issues around the globe [1]. The hepatitis B virus (HBV) is estimated by the WHO to be the 10th biggest cause of mortality globally, and 350 million people worldwide have chronic HBV infection [2]. Hepatitis B and its consequences, such as liver cancer, cause up to a million fatalities per year. Hepatitis B-related problems claim the lives of about two individuals every minute [3]. HBV infection causes a wide range of liver diseases, from acute (including fulminant hepatic failure) to chronic (including cirrhosis), and is a major risk factor for hepatocellular carcinoma [4,5].

Hepatitis B is a blood-borne disease that spreads from person to person through contact and practices that entail the interchange of body fluids, such as unsafe sex or blood transfusions from an infected person. This can happen during sex, when sharing syringes, needles, or other injectable supplies, or even when a mother gives birth to a child [6]. HBV infection is more common in low-income countries since treatment, prevention, and diagnostic options are inadequate [7]. The Pakistan Medical Research Council (PMRC) carried out a national survey from July 2007 to May 2008, which revealed that 2.5% of Pakistanis are infected with HBV [8]. The main causes of the high prevalence of HBV in Pakistan include poverty, congested population areas, lack of education, and ineffective HBV immunization methods [9].

HBV is an enveloped virus that belongs to the Hepadnavirus family [10]. HBV surface antigen (HBsAg) is a lipoprotein commonly used to diagnose HBV infection. It is a complicated structure of glycoprotein and lipid [11]. HBV laboratory diagnosis includes the identification of indicators in the serum [12]. HBsAg is the serological marker used worldwide for the diagnosis of hepatitis B. Antibodies against HBsAg are used in enzyme immunoassay (EIA) tests to gather antigen from a sample. Enzyme-linked immunosorbent assay (ELISA) is a specific, widely used type of EIA performed on a solid phase for detecting antigens or antibodies. ELISA-based detection of HBsAg is the recommended gold standard for the diagnosis of HBV infection [13].

Three HBsAg rapid tests have been found to fulfill the WHO prequalification criteria so far: VIKIA HBsAg (bioMérieux SA, Marcy-l'Étoile, France), Determine HBsAg 2 (Abbott Laboratories, Chicago, Illinois, United States), and SD Bioline WB (Abbott Laboratories). Several studies also show the high precision of these tests in determining HBsAg positivity [14]. Even though EIA tests are highly successful, they require expensive, high-quality laboratories with cold storage for chemicals, skilled personnel, and a consistent power source. In places where HBV is endemic, most of these logistics are typically in short supply [15]. 

In routine practice, ELISA testing of blood samples can be conveniently done in settings with proper laboratories and laboratory staff. However, the highly endemic areas are usually villages and places without proper healthcare facilities, which are hubs for the spread of infection, and do not have such facilities, especially in low- and middle-income countries. The development of a technique for the safe and easy transfer of a specimen from such areas to well-equipped hospitals for ELISA/EIA is hence very necessary.

A gingival swab is an easy alternative to blood sampling, and gingival crevicular fluid (GCF) is collected just by touching the swab with the gums and does not require the expertise of a phlebotomist. GCF is a serum-like fluid found in the gingival sulcus (the space between the tooth surface and the surrounding gingival tissue) [16].

Since most areas of Punjab, Pakistan, are very hot and the temperature goes as high as 50 ^o^C, we wanted to study the effect of heat on GCF samples taken by swabs. The swabs were eluted in phosphate-buffered saline (PBS) solution and were then stored at two temperatures to see their effect on the test sensitivity.

## Materials and methods

This was a non-randomised controlled study conducted to assess the effect of two temperatures on the detection of HBsAg using gingival swab specimens. The study was carried out in the microbiology lab at Shaikh Zayed Hospital, Lahore, Pakistan. Study duration was one year, from October 24, 2023, to October 24, 2024. The study was approved by the Institutional Review and Research Advisory Board (IRRAB), Technical and Ethical Review Committee (TERC), Shaikh Zayed Federal Postgraduate Medical Institute, Lahore, Pakistan (approval number: SZMC/TERC/Internal/489/2023). Informed consent was obtained from the patients. All data collected was kept confidential, and no other use of the information was made.

Study population

Inclusion criteria were patients previously confirmed as HBsAg positive and negative through standard serological testing (ELISA). Since the study was done on patients coming to the gastrointestinal outpatient department of Shaikh Zayed Hospital, those patients who were advised to have hepatitis B serology testing by the gastroenterologists were recruited for the study. The positive cases were the known cases coming for follow-up visits. Negative cases were different cases from the hospital with hepatitis B serology advised. Patients co-infected with other hepatitis viruses (HCV, HDV) or HIV, patients with oral lesions like gum bleeding, mouth ulcers, or gingivitis that could interfere with sampling, were excluded.

Sample size calculation

The sample size was estimated by using a 95% confidence interval (CI), a 5% margin of error, and an expected detection rate of HBsAg of 90% [17]. The following formula was used: 

$n = \frac{Z^{2} \, p \, (1-p)}{d^{2}}$, where n is the sample size, z is the desired level of significance, i.e., (1.96)² for a 95% CI, p is the anticipated population proportion = 0.90, d is the absolute precision or margin of error, i.e., 0.05.

Accordingly, a total of 138 known hepatitis B-positive patients and 138 known hepatitis B-negative patients were included in the study.

Study variables

The study variables included the exposure variable, i.e., temperature. Swabs were kept at two different temperatures, i.e., 27-32 °C and 50 °C. The outcome variable was the detectability of HBsAg from gingival swab specimens. Another variable was the duration of storage before testing, which was 24 hours.

Sample collection and detection

Blood samples were taken under aseptic conditions using a 5cc syringe. Gingival swab samples were collected by using an aseptic technique. Two sterilised swabs, A and B, were gently rubbed against the gingival crevicular area of each patient. A 1:10 dilution of PBS solution was made by mixing one part concentrated PBS (PBS 10%) with nine parts distilled water. The tips of both swabs were cut and immersed immediately in Eppendorf tubes containing 1 ml of PBS solution each (1 ml of solution was taken to let the swab tips be completely submerged). Eppendorf A was kept in an incubator set at 50 °C for 24 hours. Eppendorf B was kept at room temperature (27-32 °C was the room temperature in the lab) for 24 hours.

HBsAg detection was done by automated ELISA on all three samples from each patient, i.e., blood, swab A, and swab B. ARCHITECT HBsAg Qualitative II (Abbott Laboratories) was the machine used. The chemicals used are PBS solution and anti-HBs-coated microparticles in MES (2-(N-morpholino)ethanesulfonic acid) buffer with protein stabiliser.

A standardised collection method was used for all patients. Samples were coded and processed by blinded laboratory personnel to reduce observer bias. HBsAg detection was recorded as positive or negative (qualitative variable).

Data analysis

Data were analysed using IBM SPSS Statistics for Windows, version 29.0.2 (IBM Corp., Armonk, New York, United States). Descriptive statistics were expressed as percentages. A chi-square test was applied to compare detection rates across the two different temperature conditions. p < 0.05 was considered statistically significant.

## Results

**Figure 1:**
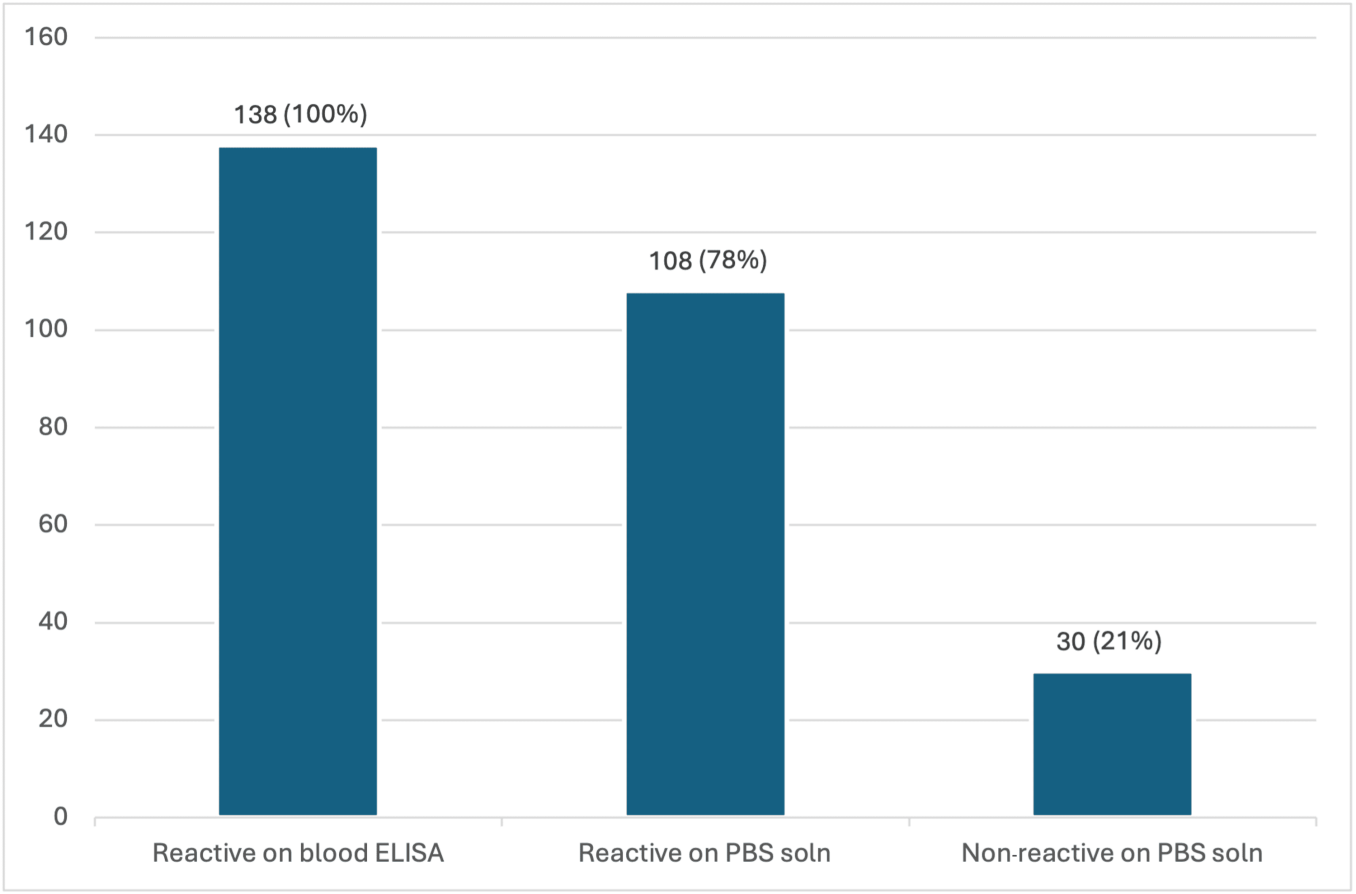
Comparison of results of ELISA done on blood samples versus PBS solution (gingival swab samples) of known HBV-positive patients It should be noted that the numbers 138, 108, and 30 refer to patient counts, rather than swab counts, as two swabs were collected per patient for testing. The y-axis in Figure 1 indicates the number of patients. The first bar in the figure depicts 138 patients whose blood samples tested reactive on ELISA, representing the entire cohort. Blood samples were not subjected to testing at two different temperatures; thus, no stratification is shown for blood sample results. The second bar reflects the number of patients (n=108) whose swab samples tested reactive by ELISA. ELISA: enzyme-linked immunosorbent assay; PBS: phosphate-buffered saline; HBV: hepatitis B virus

Out of a total of 138 confirmed hepatitis B positive patients, HBsAg was detected by ELISA performed on PBS solution prepared from swab samples in 108 patients, representing 78.26% of the cohort. Conversely, 30 patients (21.74%) tested negative on swab samples despite being positive on blood ELISA. The 30 patients testing negative on swab samples were positive on blood ELISA, indicating differences in detection sensitivity that may be influenced by sample type and processing conditions (Figure 1).

**Figure 2:**
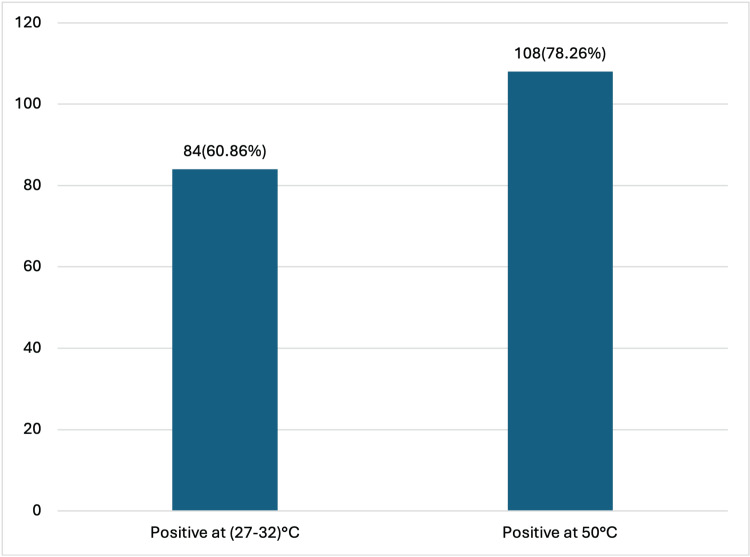
Results of ELISA done on PBS solution (swabs) kept at two different temperatures (27-32 °C and 50 °C) ELISA: enzyme-linked immunosorbent assay; PBS: phosphate-buffered saline

To see the effect of temperature, two swabs, A and B, were taken from each patient and were kept at two temperatures, i.e., 50 °C in an incubator and 27-32 °C (room temperature), respectively. Room temperature was maintained in this range with the help of an air conditioner in the lab. Of the 108 patients with positive swab ELISA results, 84 patients (60.86%) demonstrated reactivity in both swabs, i.e., those maintained at room temperature (27-32°C) as well as those stored at 50°C. An additional 24 patients (17.39%) were reactive only in the swabs kept at 50°C, whereas their paired swabs stored at room temperature yielded negative results. This shows that a greater number of swabs were positive at 50 °C as compared to room temperature (27-32 °C). Thus, while blood samples were uniformly positive, temperature variation influenced the detection rate in swab samples (Figure 2). Cross-tabulation between the two temperature results is given in Table 1.

**Table 1:** Results of known HBV-positive patients’ samples on PBS solution ELISA at two temperatures HBV: hepatitis B virus; PBS: phosphate-buffered saline; ELISA: enzyme-linked immunosorbent assay

		50 °C		
27-32 °C		positive	negative	total
	positive	84	0	84
	negative	24	30	54
	total	108	30	138

Statistical analysis

The null hypothesis was: There is no significant difference between HBsAg detection rates by ELISA at different temperatures using gingival swabs. The alternative hypothesis was: There is a difference between HBsAg detection rates by ELISA at different temperatures using gingival swabs.

The results of the chi-square goodness-of-fit test yielded a calculated chi-square statistic of 57.6 with df = 3 and a corresponding p-value of 1.89161E-12, which means 1.89161*10⁻¹², which is equal to 0.000000000000189161. Comparing this calculated value to the critical chi-square value at a significance level of α = 0.05 (critical value = 7.814 with df = 3), we find that the calculated chi-square value falls in the critical region, leading us to reject the null hypothesis.

The implications of this finding suggest that the temperature at which gingival swabs are stored or used in PBS solution can influence the reliability and sensitivity of HBsAg detection.

## Discussion

The results of the study brought some interesting facts to light. Considering the high temperatures we face during summers in Pakistan, we were concerned that this high temperature would influence the swab samples and may affect the sensitivity. Surprisingly, a greater number of swab samples came out to be positive at 50 °C as compared to the ones kept at room temperature (27-32 °C). In fact, 24 cases turned out to be reactive on the swabs kept at 50 °C only. Swabs from the same patients kept at 27-32 °C were non-reactive. Thus, we can infer that no cold chain is required to transfer these swab samples from field areas, as temperatures as high as 50 °C did not negatively impact the test's sensitivity.

Mendy et al. made similar observations in 2005 [18]. Their study on the stability of dried blood samples (DBS) revealed that the detection of HBsAg was unaffected by storing DBS for up to a month in humid conditions at temperatures ranging from 30 °C to 33°C. However, a study done in Scotland in 2015 showed some different results [19]. DBS specimens were kept in an airtight box at 70 °C, 20 °C, 4 °C, 22-28 °C (room temperature), and 37 °C. DBS eluates were also made with DBS elution buffer (PBS Tween (0.05%)) and stored at -20 °C or -70 °C. DBS eluates and cards were checked for HBsAg on day 0 and at 14, 70, and 200 days. Abbott Architect was used to detect HBsAg. DBS specimens stored at 37°C showed a decrease in HBsAg reactivity after 14 days; no loss was shown in HBsAg detection in eluates stored at -20 °C or -70 °C, but an average increase in the reactivity of 10.6% and 21.4%, respectively, from day 0 was detected [19].

HBV is quite common in Pakistan, with an estimated 9.8 million people infected by 2020 [20,21]. The number of new HBV cases is expected to rise if control measures are not adequately implemented, with a large increase predicted by 2030 due to continued transmission and low treatment coverage [21]. The reasons could be a lack of sufficient health facilities, a poor economic situation, and a lack of public understanding about the transmission of infectious illnesses such as HBV [22]. Prompt diagnosis is hence very important. The cost impact of HBV infection is significant [23]. It is essential to screen infected people to identify chronically infected sources who can develop life-threatening complications of chronic liver disease and spread the virus to others, thus raising the disease's incidence [24].

The stability of HBV markers in DBS has previously been studied [25-28]. However, the findings are inconclusive, and no research has been done on the long-term preservation of DBS eluates for HCV and HBV analysis. It is very easy to take swab samples from the gingival crevicular area and transfer them to labs with ELISA facilities. Further studies can be done to improve the technique, and it can greatly help in the diagnosis of hepatitis B in rural areas with no labs.

The study had certain limitations. It was a single-centre, non-randomised study, limiting generalisability. Only two temperature ranges (27-32°C and 50°C) were assessed, and the duration of exposure was limited to 24 hours; therefore, the effects of other temperature levels, longer storage times, or combined environmental factors (e.g., humidity) were not evaluated. Moreover, HBsAg detection by swab was reliable only when the signal-to-cutoff (S/CO) ratio in blood samples was ≥1000 on ELISA, meaning patients with low antigen levels may yield false-negative results.

## Conclusions

This study found that storage temperature has a substantial effect on HBsAg detection from gingival swabs. Incubation at 50°C produced more positive findings than at room temperature, implying that regulated heat may increase antigen release and sensitivity. Gingival swabs can thus be a reliable non-invasive alternative to blood samples if stored and transported properly. Nonetheless, validation with bigger cohorts, numerous assay platforms, and different storage times is required before routine clinical application.
